# Cystoscopy Accuracy in Detecting Bladder Tumors: A Prospective Video-Confirmed Study

**DOI:** 10.3390/cancers16010160

**Published:** 2023-12-28

**Authors:** Cathrine Silberg Guldhammer, Juan Luis Vásquez, Vibeke Møllegaard Kristensen, Thomas Norus, Naomi Nadler, Jørgen Bjerggaard Jensen, Nessn Azawi

**Affiliations:** 1 Department of Urology, Zealand University Hospital, Sygehusvej 10, 4000 Roskilde, Denmark; tdf283@alumni.ku.dk (C.S.G.); julv@regionsjaelland.dk (J.L.V.); vibek@regionsjaelland.dk (V.M.K.); tpno@regionsjaelland.dk (T.N.); naon@regionsjaelland.dk (N.N.); 2 Department of Clinical Medicine, University of Copenhagen, Noerregade 10, 1165 Copenhagen, Denmark; 3 Department of Urology, Arhus University Hospital, 8200 Aarhus, Denmark; jb@skejby.net

**Keywords:** bladder cancer, cystoscopy, surveillance program, sensitivity, specificity, overdiagnosis, overtreatment, aggressive malignant lesions, supporting methods, urine cytology

## Abstract

**Simple Summary:**

This study investigates the accuracy of cystoscopy, a key diagnostic tool for bladder cancer, which is the 10th most common cancer worldwide. Conducted at Zealand University Hospital from July 2021 to November 2022, the research analyzed video recordings of cystoscopies performed on 565 patients. The goal was to assess how well cystoscopy can detect bladder tumors in patients already diagnosed with cancer. This study found a concerning gap in the effectiveness of cystoscopy: of 181 patients initially showing no signs of cancer, 17 (9.4%) were later diagnosed with bladder cancer. This result indicates that cystoscopy has a sensitivity of 81% and a specificity of 73%, suggesting a notable risk of missing or underdiagnosing bladder tumors. These findings underscore the need for additional diagnostic methods alongside cystoscopy to improve accuracy and patient care. This study’s conclusion is pivotal for public health, as it highlights a significant issue in bladder cancer surveillance and the necessity for more comprehensive diagnostic strategies to ensure timely and effective treatment for patients.

**Abstract:**

Background: Bladder cancer ranks as the 10th most common cancer globally. The diagnosis of bladder tumors typically involves cystoscopy. Objective: This study aimed to evaluate the sensitivity and specificity of cystoscopy in detecting bladder tumors within a surveillance program following a bladder cancer diagnosis. Design, Setting, and Participants: This study utilized recordings of cystoscopies conducted at the Department of Urology, Zealand University Hospital, between July 2021 and November 2022. Clinical observations were cross-referenced with pathological results or follow-up cystoscopies. Clinically negative cystoscopies were further scrutinized for potential overlooked tumors. Outcome Measurements and Statistical Analysis: Sensitivity and specificity of cystoscopy were assessed through ROC curve analysis. Results and Limitations: A total of 565 cystoscopies were recorded, with 135 indicating clinical positivity. Among 181 cystoscopies with clinically negative results that underwent a follow-up cystoscopy, 17 patients (9.4%) were subsequently diagnosed with bladder cancer, with the lesions identified in the initial cystoscopy. The sensitivity and specificity of cystoscopy in these cases were 81% and 73%, respectively. Conclusion: This trial underscores the underdiagnosis and undertreatment of bladder tumors within the current surveillance program. Additionally, aggressive malignant lesions may be overlooked, heightening the risk of disease progression. Therefore, it is recommended that cystoscopies be complemented by other diagnostic methods to ensure accurate diagnosis and proper patient treatment. Patient Summary: This study involved 316 patients who underwent video-recorded cystoscopies and subsequent follow-up. Of these patients, 181 initially exhibited no clinical signs of bladder cancer. However, upon reviewing the recorded cystoscopy, bladder cancer was identified in 17 patients (9.4%).

## 1. Introduction

The treatment options for bladder cancer depend on various factors, such as the stage and grade of the tumor and the overall health of the patient. Non-muscle-invasive bladder cancer (NMIBC) can often be treated with transurethral resection of the bladder tumor (TURBT) and intravesical therapy with chemotherapy or immunotherapy to reduce the risk of recurrence [1]. In contrast, more aggressive treatment options, such as radical cystectomy or chemotherapy and radiation therapy, are typically required for muscle-invasive bladder cancer (MIBC) [2].

Approximately 75% of patients with BC present with disease confined to the mucosa (stage Ta, carcinoma in situ [CIS]) or submucosa (stage T1). In younger patients (<40 years), this percentage is even higher [3]. Patients with TaT1 and CIS have a high prevalence due to long-term survival, in many cases with multiple recurrences, but lower risk of cancer-specific mortality (CSM) compared to T2–4 tumors [4,5].

Despite the advances in diagnosis and treatment, bladder cancer continues to pose a significant health concern, given the high rates of recurrence and the potential risk of progression. Hence, regular surveillance is vital for early detection of recurrent or new tumors in NMIBC patients. The standard of care for surveillance involves cystoscopic examinations every 3–6 months during the first year and less frequently thereafter [6]. However, cystoscopy has limitations, and researchers are exploring alternative approaches.

One promising approach involves using optical imaging techniques to improve the visualization of bladder tumors during cystoscopy. Narrow-band imaging (NBI), a type of optical imaging that enhances the contrast between the tumor and surrounding tissue, can improve the detection and characterization of bladder tumors [7]. Other techniques, such as blue-light cystoscopy and confocal laser endomicroscopy, are also under investigation for their potential to enhance the accuracy of cystoscopic examinations [8]. However, the effectiveness of these techniques is contingent upon the cystoscopist’s proficiency in interpreting the findings. Artificial intelligence (AI) methods, e.g., CNN, have been criticized for being block-box technologies that produce oracle answers such as “positive” and “negative” without any explanation [9].

Surveillance of cancer patients for disease recurrence is essential in the management of the disease. In the case of urinary tract cancer, both urinary cytology and cystoscopy are important tools for follow-up. Cystoscopy, in particular, is critical for detecting and diagnosing recurrence and making prognostic assessments following TURBT. However, despite the importance of cystoscopy, recurrence rates after TURBT remain high, with 50–70% of patients experiencing recurrence, and approximately 13% of recurrences being detected during the first cystoscopy [10,11]. Given the significant role that cystoscopy plays in follow-up, its specificity is crucial, as overlooking lesions may have significant consequences.

The objective of this study was to evaluate the quality of cystoscopy objectively by recording and reassessing the cystoscopy in both white light and narrow-band imaging (NBI).

## 2. Methods

This study was designed as a prospective observational investigation conducted at the Department of Urology at Zealand University Hospital from July 2021 to November 2022. The participants included individuals under investigation for macroscopic hematuria or those with a previous diagnosis of bladder cancer referred to the surveillance cystoscopy program. All participants provided consent for recording their cystoscopies for educational and research purposes. Patients with recorded cystoscopies were included, while those with clinically negative cystoscopy without a control cystoscopy during data collection were excluded.

The cystoscopy groups were defined as follows:

1. True negative: Cases with clinically negative cystoscopy during video recording and a negative control cystoscopy.
2. True positive: Cases with clinically positive cystoscopy during video recording, confirmed by pathology indicating bladder tumor.
3. False negative: Cases with clinically negative cystoscopy during video recording, but positive control cystoscopy with positive pathological findings. Video review of the original cystoscopy was found clinically positive.
4. False positive: Cases with clinically positive cystoscopy during video recording, but negative histology.

Three independent urologists, blinded to clinical and pathological findings, evaluated the cystoscopy videos. Each reviewer, trained and experienced in interpreting cystoscopy videos, used the European Urological Bladder Diagram to guide evaluations. Reviewers identified suspicious lesions or abnormalities in the bladder, including discoloration, papillary lesions, and irregularities in the bladder wall.

Particularly, videos of patients with clinically negative cystoscopies but subsequently diagnosed with bladder cancer through pathological examination were meticulously reviewed. Reviewers examined the videos for possible missed tumors or other indications of bladder cancer.

A systematic review process was implemented, dividing the bladder into ten locations, further categorized into eight groups based on anatomical location and clinical significance. Reviewers evaluated each location individually, noting findings according to predefined categories.

In addition to location-based review, reviewers provided an overall impression and overview of the cystoscopy videos, considering factors like image quality, presence of artifacts or distortion, and the overall appearance of the bladder.

To assess the accuracy of cystoscopy videos, strict criteria were applied for determining false-negative results. Specifically, a cystoscopy was considered false negative if at least two out of three reviewers identified a tumor missed by the original examiner during the initial cystoscopy. This rigorous evaluation process ensured accurate identification and noting of any missed tumors, contributing to the reliability of cystoscopy sensitivity and specificity in this study.

## 3. Results

**Figure 1:**
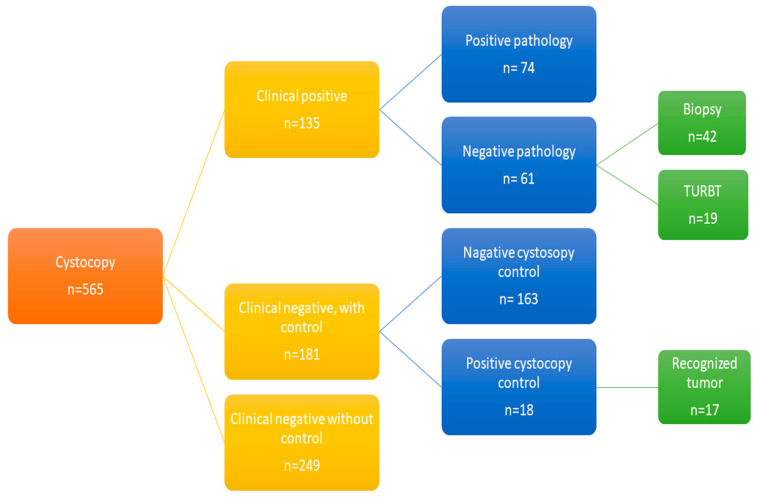
Depicting the patient inclusion process for the trial.

A total of 565 cystoscopies were recorded, with 135 cases clinically positive for bladder cancer, and 74 cases were confirmed positive by histological biopsies (Figure 1). The mean size of tumors in true-positive cases was 18.2 mm (standard deviation 25.8), and the majority (68%) of these cases were classified as Ta tumors. Tis, T1, and T2 tumors accounted for 11%, 9%, and 12% of the cases, respectively. The demographic characteristics of patients who underwent a control cystoscopy are presented in Table 1.

**Figure 2:**
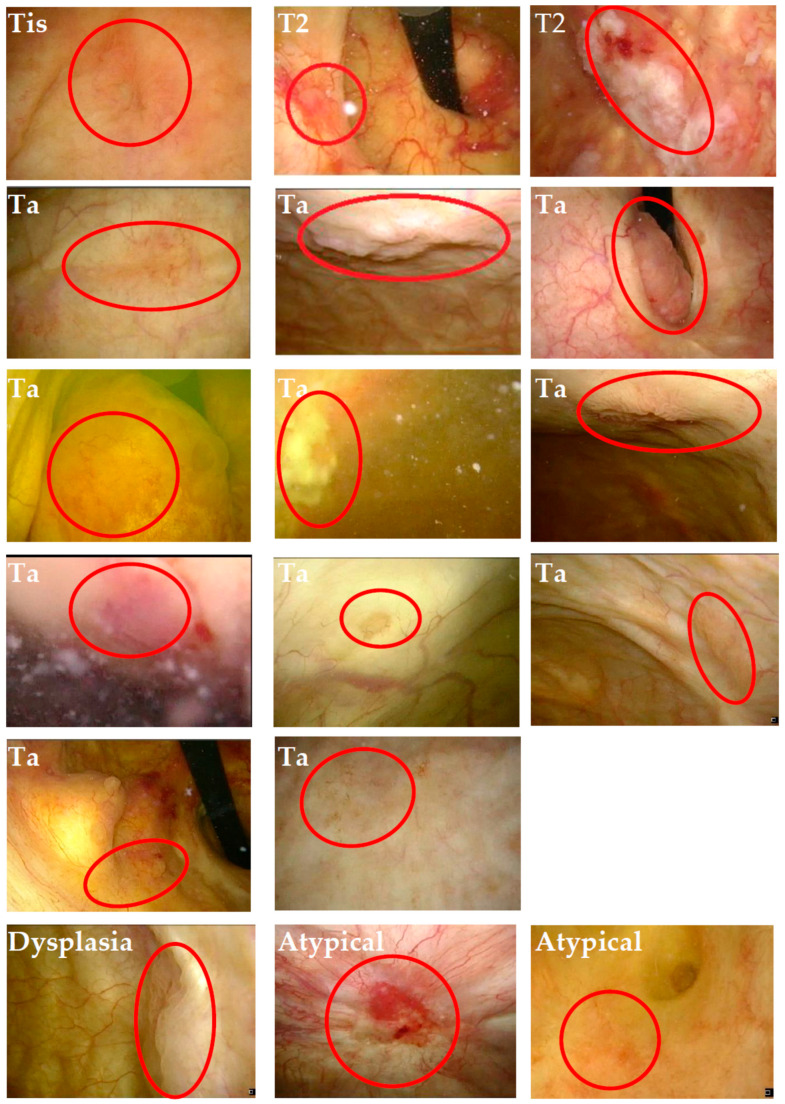
Cryptoscopic image with overlooked and annotated tumor. The red circle reveals the previously unnoticed tumor.

Of the remaining 430 clinically negative cystoscopies, 181 underwent a control cystoscopy and were included in the analysis. Among these, 18 revealed secondary abnormal tissue in the bladder with positive histology, and reviewers identified the lesions in 17 previously recorded procedures (see Figure 2 and Table 2). The calculated sensitivity and specificity of the cystoscopy examination were 81% (74/91) and 73% (163/224), respectively. The details of the seventeen false-negative cystoscopies are presented in Table 3. The mean tumor size was 9.5 mm (standard deviation 7.8 mm), the mean age was 73 years, and the female-to-male ratio was 3:14. All patients had a prior diagnosis of bladder cancer, had undergone radical treatment, and were under surveillance in the control program.

Out of the 565 recorded cystoscopies, 61 were false positive, leading to patients undergoing TURBT and/or biopsy. Eleven patients experienced adverse events, including macroscopic hematuria, cystitis, pain, retention, perforation, and one requiring a blood transfusion after the procedure. Table 4 provides a detailed overview of these cases.

Overall, compromised overview was observed in 18% (3/17) of the cystoscopies, and 29% (5/17) were deemed unsystematic.

## 4. Discussion

Our comprehensive study revealed that 9.4% of cystoscopies initially deemed clinically negative culminated in false-negative diagnoses, and subsequent diagnoses of bladder tumors were made after reviewing the recorded videos This finding raises substantial clinical concerns, particularly considering that a notable 18% of these false-negative cases were later classified as either MIBC or CIS. Both MIBC and CIS are recognized for their aggressive nature and are closely associated with an elevated risk of disease progression and metastasis. Additionally, the remaining false-negative cases were diagnosed with NMIBC. Although NMIBC is generally considered less aggressive compared to MIBC or CIS, it still necessitates careful surveillance due to its potential, albeit lower, risk of progression.

It is noteworthy that our study exclusively included patients with a control cystoscopy to validate the recorded cystoscopy. This observation underscores the possibility of overlooked lesions during cystoscopies. Importantly, our study design focused on patients who underwent a control cystoscopy, highlighting a potential gap in diagnostic protocols. Specifically, patients under investigation for first-time macroscopic hematuria typically undergo an initial cystoscopy and CT urography. If the results are negative, further follow-up controls are not routinely administered, increasing the risk of overlooking aggressive bladder cancer diagnoses. This emphasizes the importance of comprehensive diagnostic strategies to minimize the risk of missed aggressive bladder cancer cases, especially in scenarios where patients might not receive additional follow-up examinations after an initially negative assessment.

Additionally, our study highlighted the clinical consequences of using cystoscopy in initial diagnosis and follow-up according to the original bladder cancer protocol. We found that 45% (61/135) of the clinically positive cystoscopies had no pathology in the final histological report, leading to unnecessary procedures such as TURBT in 31% (19/61) of cases. TURBT is a treatment method associated with a 16% incidence of postoperative complications, such as hematuria (51%), acute urinary retention (23%), and in rare cases, extra-peritoneal bladder perforation (1.45%) [12]. Our study identified similar complications, including macroscopic hematuria, cystitis, retention, and perforation. This overtreatment imposes significant costs on both the patients and the healthcare system, including hospitalization, sick days, anesthesia, catheterization, pain management, and medication. These costs could have been avoided with a more accurate investigation, thereby reducing the unnecessary burden on hospital wards and surgical sections.

The sensitivity of cystoscopy in our study was found to be 81%, which is consistent with the reported range in the literature of 68–100%. A systematic review also reported a specificity range of 57–97%, with our study reporting a specificity of 73% [13]. However, one study showed a lower specificity than reported in other studies [14]. It is important to note that many studies report high specificity, but their inclusion criteria and evaluation methods differ from our study, which evaluated cystoscopy using a systematic review of recorded video and did not rely solely on biopsies [15]. A limitation of our study is that overlooked tumors would not have been biopsied, and the pathology does not cover the entire bladder’s histology.

In another study, cystoscopy specificity was compared to ultrasound and cytology [16]. Ultrasound was found to have limited specificity and cannot be used as a reliable reference. Cytology, on the other hand, has low sensitivity, particularly in low-grade NMIBC.

The accuracy of cystoscopies can be affected by various factors, such as insufficient overview, macroscopic hematuria, fibrin, infection, and small tumor size, leading to low specificity. Therefore, it is crucial to perform systematic cystoscopies to minimize the risk of missing potential tumors. Our study revealed compromised systematic overview in 18% (3/17) of cystoscopies, emphasizing the need for additional supporting methods. AI is an emerging technology in the medical field, which has already demonstrated its success in the diagnosis of skin cancer, gastric cancer, and other cancers [17,18]. However, there are limited studies on the potential application of AI in urologic cystoscopies [19].

Flexible cystoscopy is a key competency in diagnostic urology. Most follow-up cystoscopies in the Department of Urology at Zealand University Hospital are performed by junior urology residents or trained urology nurses. The Halstedian era of “see-one, do-one, teach-one” in medical education is moving towards more mastery based learning, where novices must demonstrate what they have learned and pass a test before. Numerous simulation platforms are available, and are being integrated into the curriculum of Urology residency all over the world. Multiple studies have shown that simulation-based training before clinical training can lower the learning curve for procedure novices and thereby improve the skills of future surgeons and procedurists [20,21]. Furthermore, all residents in our department must pass a simulation based cystoscopy test and two days of supervised cystoscopies before they proceed to performing independent surveillance cystoscopies in the out-patient clinic. Maintenance and testing of the acquired skills are not evaluated after the initial pass/fail test. Our study may also indicate that we do not maintain or improve the skills of novices after initial testing and an annual test might be sensible to implement.

Bladder cancer diagnosis can be challenging, and cytology has limited sensitivity in detecting bladder tumors. It is only helpful in identifying high-grade tumors and cases of high-grade malignancy or CIS. Unfortunately, there are no reliable urinary markers currently available that can specifically diagnose either MIBC or NMIBC. However, commercially available urine biomarkers are being studied for their usefulness in diagnosing recurrence during NMIBC follow-up. Further research is necessary to determine their accuracy and effectiveness, but they may become important tools in the diagnosis and surveillance of bladder cancer [22] potentially offering reduced discomfort and inconvenience for patients.

The specificity of CT urography in detecting bladder tumors can vary depending on the type of tumor. For instance, CIS, which is a flat mucosa lesion without any papillary mass protruding into the lumen, cannot be accurately detected by CT urography according to several studies [23,24].

However, a study reported that while CT urography failed to identify CIS, it was successfully detected by cystoscopy. Conversely, in cases of macroscopic hematuria, CT urography exhibited high sensitivity (100%) and specificity (95%), showing substantial concordance with tumor detections made through cystoscopy [25]. Therefore, the use of CT urography in the diagnosis of bladder tumors may vary depending on the type of tumor and the presenting symptoms. Additional research is necessary to determine the accuracy and effectiveness of CT urography in diagnosing different types of bladder tumors.

Assessing the sensitivity and specificity of cystoscopy presents a notable challenge, given the absence of a universally accepted gold standard in this context. Furthermore, differentiating between recurrent lesions and previously overlooked lesions can be a complex task. To address these challenges, this article adopted a strategy involving video recordings to enhance the precision of the procedure’s evaluation.

The utilization of video recordings allowed reviewers to witness the identical conditions and details of the examination as the original examiner. This methodology not only guaranteed consistency and reliability but also mitigated potential discrepancies, thereby enhancing the overall accuracy of the findings. By leveraging video recordings, this study aimed to overcome the inherent limitations associated with assessing cystoscopy efficacy, providing a more nuanced and reliable evaluation of the procedure.

### Improvement in Bladder Cancer Detection

In the ongoing effort to improve bladder cancer detection, recent advancements have introduced several innovative methods that supplement traditional cystoscopy. These include narrow-band imaging (NBI), blue-light cystoscopy (BLC), confocal laser endomicroscopy (CLE), various urine biomarkers, and AI technologies. Each of these approaches offers unique advantages in identifying bladder tumors, often difficult to detect using conventional methods.

Narrow-band imaging (NBI) significantly enhances the visualization of the bladder’s vascular patterns and mucosal textures, providing a more detailed view than standard cystoscopy. It helps in distinguishing flat lesions and carcinoma in situ, which are typically hard to detect. Studies, like those by Zheng et al. [26,27], show that NBI can potentially increase bladder cancer detection sensitivity to as high as 94%, with a specificity of 85%.

Blue-light cystoscopy (BLC) involves using a photosensitizing agent that causes cancer cells to fluoresce under blue light, thus distinguishing them from healthy tissue. This technique has been particularly effective in identifying carcinoma in situ. Pederzoli et al. [28,29] found that BLC could achieve a sensitivity of 93% and a specificity of 46%, indicating its potential in improving bladder cancer detection rates.

Confocal laser endomicroscopy (CLE) provides real-time, microscopic imaging of the bladder lining during cystoscopy, allowing for the accurate differentiation between benign and malignant lesions. Wu et al. [30,31] reported that CLE could achieve a sensitivity of 90% and a specificity of 72%, underscoring its value in increasing diagnostic accuracy.

Traditional urine cytology, known for its poor sensitivity [13,32], has led to the development of various urine marker tests for bladder cancer detection. These tests, non-invasive and convenient, can be performed remotely. UroVysion, relying on fluorescence in situ hybridization (FISH), has shown high sensitivity (73%) and specificity (95%) in diagnosing bladder cancer. Other tests like Uromonitor, CxBladder, Xpert, and Bladder Epicheck use PCR (polymerase chain reaction) technology, demonstrating high sensitivity and specificity, particularly in follow-up contexts [32,33,34,35]. Bladder Epicheck, for instance, has 100% sensitivity and specificity for high-grade tumors [34]. These urine marker tests stand out for their widespread availability and ease of use, allowing for simultaneous testing of multiple patients.

AI is increasingly being used in medical diagnostics, including in areas such as skin and gastric cancer. Its application in bladder cancer detection, through machine learning and deep learning algorithms, holds the potential to overcome some limitations of current diagnostic methods, enhancing early detection and patient outcomes [17,18,19].

While these advanced techniques (NBI, BLC, CLE, urine biomarkers, AI) offer promising improvements in bladder cancer detection, they differ fundamentally from traditional cystoscopy in their operational principles and implementation requirements. They often involve significant costs, specialized equipment, and training, contrasting with the more universally available and cost-effective nature of traditional cystoscopy. Moreover, the effectiveness of urine biomarkers varies depending on the context, and AI applications in bladder cancer detection are still in their early stages. These factors, along with the inherent limitations of human interpretation that can introduce errors, highlight the challenges in directly comparing these advanced methods to findings from studies like ours, which focus on traditional cystoscopy. Our study, grounded in a systematic review of recorded cystoscopy videos, offers insights immediately relevant to a broader range of clinical practices, emphasizing the practicality and applicability of traditional cystoscopy in current healthcare settings.

## 5. Conclusions

Based on our trial, it is evident that the existing surveillance program for cystoscopies may result in the overdiagnosis and treatment of some non-aggressive lesions, while concurrently overlooking more aggressive malignant lesions. Consequently, there is a compelling need for the integration of supplementary methods to augment cystoscopies and ensure accurate treatment, especially for patients whose surveillance program might be terminated based solely on cystoscopy results.

Enhancing the specificity and accuracy of cystoscopies is imperative for minimizing the unnecessary treatment of patients with non-aggressive lesions and, concurrently, ensuring appropriate treatment for those with more aggressive malignant lesions. Future research endeavors should continue to explore the potential of adjunctive methods, such as AI and urine biomarkers, to augment the precision of bladder cancer diagnosis and follow-up procedures. This approach is essential for advancing the effectiveness of diagnostic protocols and optimizing patient care in the realm of bladder cancer management.

## Figures and Tables

**Table 1:** Patients demography.

	Total	%
**Sex**		
Male	221	76
Female	70	24
**Age**		
Mean	73.8	
**Tobacco**		
Present	71	24
Previous	132	45
Never	88	30

**Table 2:** Reviewer assessment.

Objective Assessment		Reviewer 1	Reviewer 2	Reviewer 3	%
Number of cystoscopies		17	17	17	
Approved overview					
Yes		14	15	13	82.4
No		3	2	4	17.6
The whole bladder visualized					
Yes		13	11	13	72.5
No		4	6	4	27.5
Tumor recognizes					
Yes		16	15	15	90.2
No		1	2	2	9.8

**Table 3:** Patient demography for false-negative cases.

Criteria		N (Mean)	%	SD
**Gender**				
Male		14	82	
Female		3	18	
**Age**		(72.6)		8.31
**The time between negative and positive cystoscopy**				
Mean (mdr.)		9		3.24
**Pathology T-stage**				
Ta		12	65	
Tis		1	6	
T2		2	12	
Other		3	18	
**Mean tumor size (mm)**		(9.5)		7.82

**Table 4:** Reported complication related to TURBT or biopsy.

Complication	Cases (n)
One or more	11
Macroscopic hematuria	8
Cystitis	2
Pain	2
Retention	1
Perforation	1
Blood transfusion	1

## Data Availability

The data presented in this study are available in this article.
